# An Important and Dangerous Surgical Operation

**Published:** 1851-11

**Authors:** 

**Affiliations:** At the Necker Hospital in Paris


### WESTERN JOURNAL

OF

### MEDICINE AND SURGERY.

NOVEMBER, 1851.

Art. I.—*An Important and Dangerous Surgical Operation.* By M. Lenoir, at the Neckar Hospital in Paris. Translated by P. K.

*An Important and Dangerous Surgical Operation.*—A friend who has enjoyed excellent opportunities for observing the revelations of the Hospitals of Paris, and who has turned those opportunities to good advantage, permits us to copy the following interesting case from his notes. His *porte-feuille* is rich with many cases of great interest, and we hope to have frequent opportunities of letting the raeders of the *Western Journal of Medicine* read them.—*Ed. West. Jour. of Med. & Surg.*

This morning, April 10th, 1844, M. Lenoir, at the Neckar Hospital in Paris, performed one of the most important and dangerous operations in surgery. Its subject was a lad fifteen years of age, and of lymphatic temperament, but no signs, either rational or physical, of tuberculous deposit, were afforded. Of his previous history I know nothing. At present his left lower limb is from the hip to the ankle what might, truly be called a mass of corruption. Over the ankle joint are several fistulous openings; on the posterior border of the inferior fourth of the fibula is the artificial opening of an abscess; and on the thigh there are several other similar openings. All of these are in a gangrenous state, and are secreting abundantly a brownish, fœtid, gangrenous fluid. Various means have been tried to sustain the strength of the patient, and to change the character of the disease, but all have failed, the disease has gone on from bad to worse, and the lad now seems to hold on to life by the most feeble ties. It was the intention of M. Lenoir to amputate the thigh, and this morning the patient was placed for that purpose upon the table, but then there was observed, what had not existed some hours previously, or, if it had, what had previously escaped notice, an abscess seated upon the dorsum of the illium, and pointing over the great trochanter. Into this abscess the surgeon plunged a bistoury and evacuated a large amount of ill-digested pus.

It then became not a question of amputation, but of disarticulation of the hip-joint. An operation, which under the most favorable circumstances, is sufficient to cause hesitancy on the part of the boldest, and which in this case was almost certain to be followed by death. But then death sure and speedy, would be the result should the boy be left to nature, or the usual remedies, which had already failed to produce the slightest amelioration. Now it was, that M. Lenoir exhibited the highest attributes of the skillful surgeon, quickness of thought and promptness of decision—he hesitated but for a moment, and determined to risk all on the disarticulation of the hip-joint. The operation was performed with a degree of skill and quickness that would reflect credit upon any one. In six seconds from the first prick of the knife, the limb fell to the ground.

What will be the result of this scarcely admits of a doubt; indeed, it will be wonderful if the patient is not dead now whilst I am making this note.

April 12th.—The patient survived the operation but four hours. The antopsy showed the abdominal and thoracic viscera to be in a healthy condition. The only lesion found was an abscess not suspected previous to death, seated in the right hip-joint. No clots had formed in the ligatured vessels.

Hotel Dieu, December 5, 1843.

No. 49, Salle Ste Marthe is to-day occupied by a young man, aged 21 years, strong, well built, and who has hitherto enjoyed good health, as have his parents, who are both at this present living.

About six months since he experienced, for the first time, a dull, heavy pain, tolerably constant but of intermittent intensity, occupying the right side of the head, about where the parietal and temporal bones join.

About 15 days after the first appearance came the painful sensations, upon passing his finger over the affected region he discovered a deficiency in the bone, or as he described it, a hole the size of a five cent piece. This has kept on increasing, as have the pains, and at the same time, there has been forming a tumor, the base of which corresponds to the destruction of ossific formation. The tumor is now as large as a turkey's egg, soft, fluctuant, and slightly yielding, I think, to pressure (having no authority to try experiments, I am afraid to press as long and as steadily as I could wish,) around its base there can be distinctly felt the edges of the absorbed bone, and upon its most dependent part the skin is thinned, reddened and pointing to the extent of a quarter of an inch. The health of the patient has been good since the first appearance of this affection. There have as yet been no apparent symptoms of compression, no diminution of sense, sensation, or control of muscular action.

Dec. 7th.—M. Roux on yesterday saw the patient, and deciding that it was a case of abscess with caries, laid open the tumor for the purpose of evacuating the pus. Upon cutting into it, the pus flowed abundantly, but no deficiency was to be found in the bone. In a word, the bone was perfectly healthy. The deception occurred, by the surrounding cellular tissue being unusually indurated, an effort on the part of nature to prevent the extension of the tumor.

